# A Newly Developed Online Peer Support Community for Depression (Depression Connect): Qualitative Study

**DOI:** 10.2196/25917

**Published:** 2021-07-12

**Authors:** Dorien Smit, Janna N Vrijsen, Bart Groeneweg, Amber Vellinga-Dings, Janneke Peelen, Jan Spijker

**Affiliations:** 1 Depression Expertise Center Pro Persona Mental Health Care Nijmegen Netherlands; 2 Behavioural Science Institute Radboud University Nijmegen Nijmegen Netherlands; 3 Department of Psychiatry Donders Institute for Brain, Cognition and Behaviour Radboud University Medical Center Nijmegen Netherlands; 4 Dutch Depression Association Amersfoort Netherlands; 5 Interuniversity Center for Social Science Theory and Methodology Department of Sociology University of Groningen Groningen Netherlands; 6 Research Group for Key Factors in Youth Care HAN University of Applied Sciences Nijmegen Netherlands

**Keywords:** depression, online peer support community, internet support group, experiential knowledge, self-management, empowerment, qualitative research, patients’ perspectives, participation style

## Abstract

**Background:**

Internet support groups enable users to provide peer support by exchanging knowledge about and experiences in coping with their illness. Several studies exploring the benefits of internet support groups for depression have found positive effects on recovery-oriented values, including empowerment. However, to date, little attention has been paid to user narratives.

**Objective:**

This study aims to capture the user perspective on an online peer support community for depression with a focus on the modes of user engagement and the benefits users derive from participation in the forum.

**Methods:**

In this qualitative study, we conducted 15 semistructured interviews with users of Depression Connect, a newly developed online peer support community for individuals with depression. Combining a concept-driven and a data-driven approach, we aimed to gain insight into what users value in our Depression Connect platform and whether and how the platform promotes empowerment. We performed a thematic analysis to explore the merits and demerits reported by users by using theoretical concepts widely used in internet support group research. In the subsequent data-driven analysis, we sought to understand the relationship between different styles of user engagement and the participants’ experiences with the use of Depression Connect. Data analysis consisted of open, axial, and selective coding. To include as diverse perspectives as possible, we opted for purposive sampling. To verify and validate the (interim) results, we included negative cases and performed member checks.

**Results:**

We found participation in Depression Connect contributes to a sense of belonging, emotional growth, self-efficacy, and empowerment. “Getting too caught up” was the most frequently reported negative aspect of using Depression Connect. The deployment and development of three participation styles (ie, reading, posting, and responding) affected the perceived benefits of Depression Connect use differentially, where the latter style was central to enhancing empowerment. “Being of value to others” boosted the users’ belief in their personal strength. Finally, Depression Connect was predominantly used to supplement offline support and care for depression, and it mainly served as a safe environment where members could freely reflect on their coping mechanisms for depression and exchange and practice coping strategies.

**Conclusions:**

Our findings shed new light on user engagement processes on which internet support groups rely. The online community primarily served as a virtual meeting place to practice (social) skills for deployment in the offline world. It also allowed the members to learn from each other’s knowledge and experiences and explore newly gained insights and coping skills.

## Introduction

The increased accessibility of the internet, together with the advantages of offline peer support [1-3], has boosted the development of internet support groups (ISGs). These ISGs enable users to provide peer support by exchanging knowledge about and experiences of coping with a physical or mental illness [4]. Given its recurrent, persistent nature [5] and the stigma associated with depression [6], people living with the disorder often search for self-help resources [7] and appear to be the most-active users of ISGs, logging in or posting the most frequently [4,8,9].

There has been much focus on the efficacy of ISGs for depression, with previous research examining clinical outcomes and providing compelling but inconclusive evidence for a reduction of depressive symptoms resulting from the engagement in mental health ISGs (MHISGs) [4,10] or depression-specific ISGs [11]. Additionally, descriptive content analysis studies [11,12-17], user survey studies [18-21], and randomized trials or randomized controlled trials (RCTs) [22-29] evaluating ISGs for depression generally present positive results on recovery-oriented values, such as personal strength and needs and experiences with (the road to) recovery [30]. For example, content analysis studies [11,12,14-17] and user survey studies [18-21] collectively indicate that engagement in a depression ISG increases a sense of social and emotional support, and RCTs and other clinical trials suggest short-term improvements in empowerment [22], reappraisal [24], and self-efficacy [26]. However, in this body of research, the user perspective has received far less attention [18]. Such a narrative perspective on associations between processes of user engagement and the perceived value of ISG use can increase our understanding of what users need to benefit from web-based depression platforms.

Recent RCTs on MHISGs indicate that high user engagement quantified in terms of the number of posts [29] or login frequencies [31] is relevant for attaining health gains [29,31]. This, however, implies that content analysis studies may be biased. Based on the 1% rule, which postulates that 1% of users contribute around 75% of all ISG posts [32,33], content analysis studies inevitably evaluate data of small groups of highly engaged users (often referred to as “superusers” or “posters”) without considering “lurkers” (users who follow discussions but seldom participate in them by posting) [34], whom we prefer to refer to as “readers.” Moreover, operationalized in quantitative terms, high user engagement does not capture its qualitative nature [35]. Research into ISG participation styles does allow such a qualitative assessment, with studies revealing very diverse styles across online health communities, including ISGs [36]. As to participation styles in MHISGs, the most highly engaged users were typified as “emotionally supportive companions” [35] and “active help providers” [37,38], whereas the less-active users tended to engage more in topics regarding experiential knowledge, disclosure, and informational support [35]. Considering depression-specific ISGs, the profiles identified included “concerned about daily living,” “information seekers” [19], and “interactive peer support” [20]. Moreover, contrary to quantitative analyses, qualitative characterizations of user engagement (eg, in terms of participation styles) have not yet explored how these relate to the users’ valuation of the benefits and drawbacks of the platforms.

Particularly enhanced empowerment appears to play a key role [16,22,39-41] in (depression) ISGs, where gains are assumed to be linked to frequent user engagement [42-45] and, possibly, particular participation styles. However, the conceptualization of empowerment lacks clarity [42,46,47], whereas the measures to chart users’ perspectives were also very diverse, both in nature and quality [42]. Based on their analysis of 17 definitions used in the literature, Cerezo et al [46] proposed the following narrow definition of *empowerment* in the context of patients with chronic illnesses such as depression: “an enabling process whereby health care professionals collaborate with patients to help them acquire knowledge and resources and whose outcome is a patient with greater ability to exercise control, manage his/her condition and to make informed decisions,” precluding peer-to-peer empowerment. Empowerment is a multifaceted concept [47] and is considered both a process and an outcome, with an intrapersonal component (“sense of control”), an interactional element (“critical awareness of the sociopolitical environment”), as well as a behavioral aspect (“community involvement”) [48,49]. Most studies evaluating effects of ISGs on empowerment focus on the intrapersonal component [50], whereas social processes in online communities are also likely to foster interactional empowerment [45]. Taken together, ISG use appears to promote different aspects of empowerment, but it remains unknown whether this is dependent on the nature of user engagement in relation to differential participation styles.

This study is part of a larger research project called “The Power of Depression” in which we seek to build on the recovery approach in mental health [30]. In a first exploratory study, we interviewed patients with recurrent and chronic symptoms of depression to gauge their experiential knowledge about coping strategies. The results suggested that gains in experiential knowledge mainly pertained to three intrapersonal factors: introspection, empowerment, and self-management strategies [51]. Subsequently, to facilitate the exchange of personal experiences, we developed “Depression Connect”— a closed, moderated platform providing online peer support for individuals living with depression. This platform, with a forum as its main feature, was created with the aid of a design thinking methodology following the Human Centered Design Kit (Radboudumc, REshape Center), in close collaboration with potential users currently dealing with depression, their significant others, and health professionals (psychiatrists, therapists, and psychology researchers). We made Depression Connect accessible for any person seeking help and support for depression, independent of their clinical and demographic characteristics.

We are in the process of evaluating the self-reported effects of Depression Connect on various aspects of empowerment in a quantitative longitudinal user survey (in preparation). In the qualitative evaluation we present here, we specifically sought to delineate the perceived benefits of Depression Connect participation by evaluating user experiences as a function of their participation styles. Considering the promotion of empowerment key to ISGs [16,22,39-41], as well as social and emotional dimensions that foster empowerment, we expected that participation in Depression Connect would affect users’ sense of empowerment differentially depending on their mode of engagement.

## Methods

### Depression Connect

The online peer support community Depression Connect was launched on June 19, 2019. It is a digital platform that offers people with depression the opportunity to (anonymously) read or exchange knowledge about and experiences with coping with depression. It can be accessed via a website hosted by the Dutch Depression Association; the national patient association plays a central role in organizing peer support facilities for this group in the Netherlands. Through their website, any person seeking help for depression has easy access to the Depression Connect community. Depression Connect was developed and is coordinated by our research group in close collaboration with the Centre of Expertise for Depression, part of the Pro Persona Mental Health Care. To recruit a clinical population for our study, we informed members of the patient association, visitors to the website, and patients receiving treatment in a Pro Persona Mental Health Care clinic about Depression Connect and our research project through presentations, email, and flyers. We also posted the launch of Depression Connect as a news item on various websites associated with mental health care. Although other ISGs for depression are available in the Netherlands, the close collaboration between specialized mental health services and the patient association is one of the main strengths of the Depression Connect platform. When moderating and coordinating the Depression Connect community, the perspectives of both health professionals and experiential experts are taken into account. Moreover, its structural embedding in the patient and professional organizations fosters topical relevance. For example, by posting news items about depression, both organizations can inspire conversations among users and serve as a reference framework inducing users to revisit the platform regularly.

Next, we outline the login procedure, guidelines for moderators, and functionalities of Depression Connect. When accessing the site, general terms and conditions for users, privacy policy, and engagement rules are displayed; this information can also be accessed from the homepage at all times. Any interested user can then sign up for Depression Connect membership. To access the content of the community, members always need to login. When they do so for the first time, they are invited to introduce themselves; this is not mandatory and anonymized profiles are allowed. However, the moderators can always access personal contact details (name and email) to reach members personally, if necessary. Subsequently, new users will see a manual explaining how to use Depression Connect. Upon posting the first message, users are welcomed by a member of the Depression Connect moderator team. In order to ensure a constructive exchange of peer-to-peer experiences, posts are screened twice a day by one of the 5 moderators. Since the focus groups informing the development of Depression Connect expressed a clear need for *peer* support without the involvement of professionals, experiential experts were recruited as moderators. At the end of their (morning or evening) shift, the moderators document peculiarities and the general atmosphere in the forum in a logbook to inform their successor. Moderators only intervene when the content discussed, or a member’s conduct, gives rise to conflicts with engagement rules. More specifically, they will act only when an urgent request for support is posted or when they identify suicidal tendencies in posts, and when rules of engagement are violated (eg, when contributors show disrespect for one another, disclose sensitive information to Depression Connect nonmembers or outsiders, share information on suicide, or share privacy-sensitive information such as names of doctors). When users exchange misinformation about depression, moderators will refer them to reliable, evidence-based sources of information. As an extra security mechanism, predetermined trigger words, which refer to a crisis situation, will automatically generate a notification in the moderators’ mailbox. Launched in mid-2019, the online community attracted an average of 88 new members a month and totaled 1374 members as of September 24, 2020, when the data for our quantitative user survey study were extracted.

**Figure 1:**
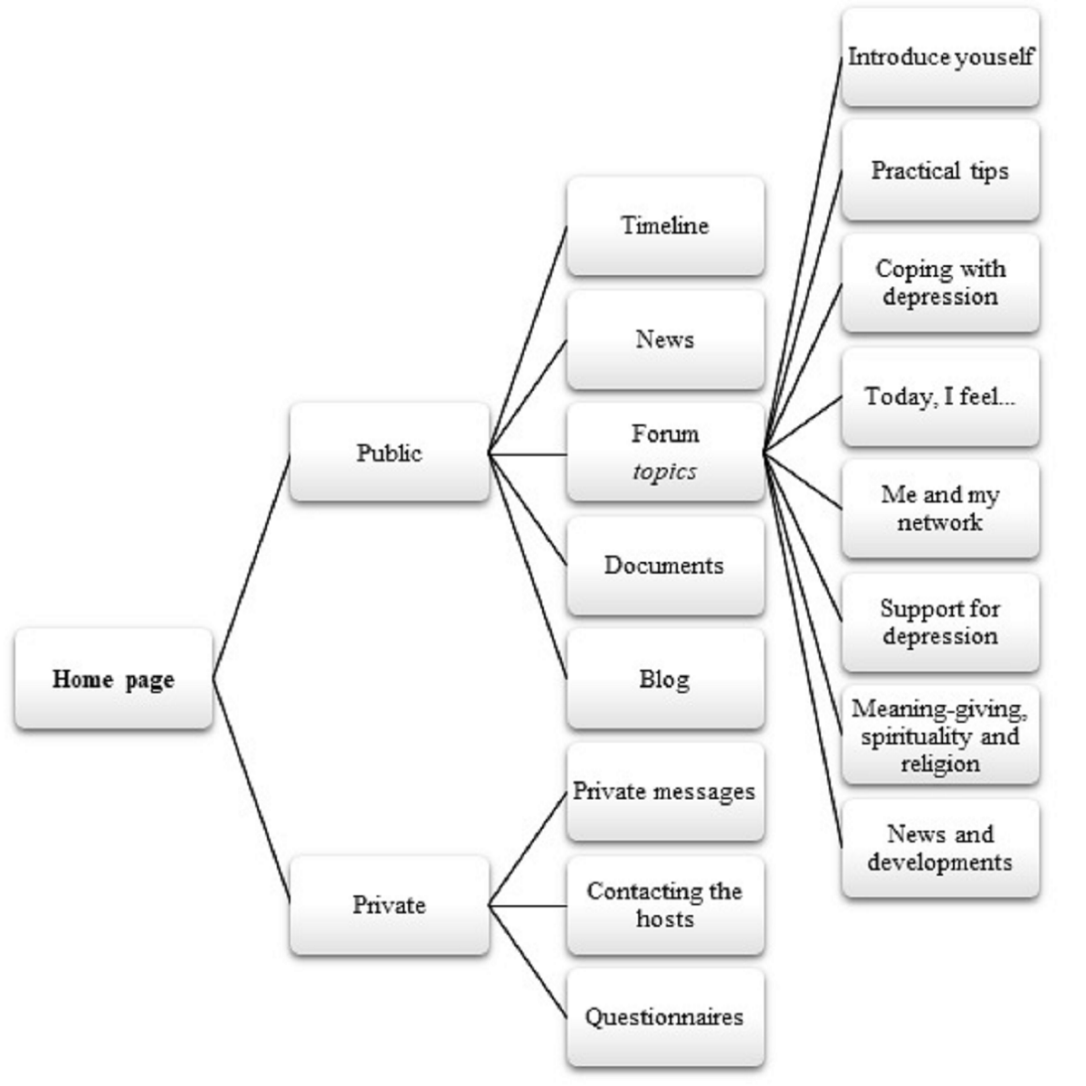
Content and structure of the online peer support community Depression Connect.

The design of the overall Depression Connect website and its forum is straightforward and user friendly, promoting positive user experiences and allowing users to navigate freely [52]. Figure 1 depicts the structural organization of the Depression Connect platform. Users can create their own topics on the forum, but we also provide eight predetermined topics that we derived from the main themes of experiential knowledge identified in our first study. Besides their contribution to the forum, members can, among other options, read news items and publications about depression (posted by the Depression Connect team), post blogs, and send private messages to other Depression Connect users.

### Study Design

In this qualitative study, semistructured interviews (see Multimedia Appendix 1 for interview guide) were conducted with Depression Connect users to explore what the online community had offered them in terms of ways to cope with their current, past, or subclinical depression. We used a hybrid approach [53] combining deductive and inductive reasoning. In the theoretical context of our larger project [51], we created a guiding framework [54] based on an inventory of experiential knowledge and the relevant literature on empowerment to deduce all relevant factors involved in the broad and complex interplay of depression ISGs [14]. We applied thematic analysis [54] to identify, examine, and gain insight into the patterns of predetermined themes. Next, using an inductive, data-driven approach based on the grounded theory [55] and Strauss’ exposition of the core principles of qualitative research in social sciences [56], we kept an open mind to avoid excluding potentially relevant observations. We used specific guidelines to analyze the data; these included open, axial, and selective coding and matrices [57], as well as tree diagrams [58], drawn from the grounded theory. This comparative and iterative approach enabled us to simultaneously analyze and gather new data to further explore and integrate concepts emerging during data collection, which continued until no new main themes emerged.

By combining these top-down and bottom-up approaches, we sought not only to learn what Depression Connect users do and do not appreciate about the platform (charting both differences and similarities among users) but also to further study the role of ISGs in developing experiential knowledge in general and empowerment in particular to (in)validate existing theories. The outcomes would complement our quantitative companion study of the effectiveness of the Depression Connect community regarding empowerment (and other aspects). In this study, all Depression Connect users were invited to complete questionnaires 3 days after enlisting, with two follow-up assessments at 3 and 6 months.

After having evaluated the research protocol in accordance with the Dutch Medical Research (Human Subjects) Act, the local ethics committee (Commissie Mensgebonden Onderzoek Arnhem-Nijmegen) waived ethical approval given the minimal burden to the study participants. All participants were asked to provide written, informed consent prior to the interview following the Declaration of Helsinki.

### Study Participants

We posted three calls for participation in our study over a 3-month period in the news section of the Depression Connect platform. Eight potential participants responded. We sent them an information letter by email, inviting them for a telephone screening. During this call, the researcher provided the candidate with a brief introduction to the study and information on the purpose of the interview, explaining the voluntary nature and confidentiality of their participation. The candidates’ demographic and clinical characteristics and patterns of use of the online community were assessed to ensure diversity within the sample. In order to obtain as wide a range of user perspectives as possible, we adopted lenient inclusion and exclusion criteria, resulting in all 8 candidates being included, with sufficient differences in characteristics and backgrounds.

After an initial analysis of these first eight interviews to derive the concepts discussed, we used purposive sampling to identify new participants with different profiles and uncover any additional themes. Members with (prior) experience in offline peer communities organized by the Dutch Depression Association were contacted by their regional coordinator, which yielded 1 participant. At this point, the research sample (n=9) solely consisted of individuals with recurrent or chronic depression. Therefore, a member who had newly joined Depression Connect, introducing herself as having been recently diagnosed with depression, was invited to participate via a personal email. We wanted to also include negative cases, that is, Depression Connect users with experiences or perspectives who were likely to deviate from other users and the main theories or evidence on ISG [59], to potentially provide unexpected findings that might ultimately strengthen the theory. Hence, we recruited 2 participants who distinguished themselves by their minimal or nonuse of Depression Connect after joining the platform. One of these participants enrolled himself upon our invitation, identifying himself as a Depression Connect member who mainly engaged in other online fora about depression. The second (female) participant was a former Depression Connect user randomly selected from a contact list of unsubscribed members who was invited via email. Recruited through a fourth and final call for participation on the platform, another 3 participants were interviewed to achieve data saturation, resulting in a final sample of 15 (former) Depression Connect members.

Table 1 shows the demographic and clinical characteristics of the study participants and the frequency and duration of their use of Depression Connect. All 15 participants had received some form of psychological care or treatment at an earlier stage in their lives, with 10 (67%) receiving current and 3 (20%) awaiting treatment for their depression (including 1 negative case); 2 (13%) participants (including 1 negative case) were not being treated at the time of the interview. Furthermore, 12 of 15 (80%) participants were taking or had taken psychotropic agents for their depression. The majority (11/15, 73%) visited the Depression Connect forum regularly, with a frequency varying from daily to once a week, barring, by definition, the 2 negative cases and 2 other members who joined the forum only irregularly.

**Table 1:** Demographic and clinical characteristics of study participants (N=15) and their engagement on the Depression Connect online support community.

Demographic characteristics					Value	
Age (years), mean (SD)					49 (11)	
**Gender, n (%)**						
	Male			6 (40)		
	Female			9 (60)		
**Ethnicity, n (%)**						
	Caucasian of Dutch descent			15 (100)		
**Educational level, n (%)**						
	Secondary education (middle or high school)			2 (13)		
	Secondary vocational education and training			7 (47)		
	Advanced vocational education and training and academic education			6 (40)		
**Clinical characteristics, n (%)**						
	**Current mental health care or treatment**					
		Intake or waiting list		3 (20)		
		**Ongoing**		10 (67)		
			Mental-health nurse practitioner (general practice)	3 (20)		
			Psychologist or psychotherapist (secondary care)	7 (47)		
		None		2 (13)		
	**Treatment history^a^**					
		Secondary mental health care (eg, CBT^b^, psychotherapy)		15 (100)		
		Previous psychopharmacological treatment		6 (40)		
		Current psychopharmacological treatment		8 (53)		
		Never used psychotropic medication		1 (7)		
	**Number of depressive episodes^c^**					
		1		1 (7)		
		2		1 (7)		
		3-5		5 (33)		
		Chronic course only (symptoms persisting ≥2 years)		7 (47)		
		Chronic course in addition to depressive episodes		3 (20)		
	**Age at depression onset (in years; range: 8-57)**					
		<12		1 (7)		
		12-18		3 (20)		
		19-32		6 (40)		
		33-45		4 (27)		
		>46		1 (7)		
	**Duration since onset (in years; range: 5-47)**					
		0-10		6 (40)		
		11-20		2 (13)		
		21-30		2 (13)		
		31-40		2 (13)		
		41-50		3 (20)		
**User engagement**						
	**Frequency of using Depression Connect, n (%)**					
		Daily		6 (40)		
		3 times a week		4 (27)		
		Once a week		1 (7)		
		Irregular		2 (13)		
		Unsubscribed after 1 month of forum use (negative case)		1 (7)		
		Inactive (negative case)		1 (0.7)		
	Duration of use (in months; excluding negative cases; range: 1.5-11), mean (SD)			6.8 (3.8)		

^a^Includes overlap in different forms of treatment.

^b^CBT: cognitive behavioral therapy.

^c^Includes overlap in chronic course and depressive episodes.

### Data Collection

From February 2020 until June 2020, two authors (DS and AD) individually conducted semistructured interviews (n=9 and n=6, respectively) with 15 Depression Connect users (including 1 former user), lasting 28.37 to 66.16 minutes (mean 48.5, SD 11.25 minutes). Both authors have a master’s degree in social sciences and are specifically trained and experienced in qualitative research methods. They had created a topic list, building upon the first exploratory study [51], the existing literature, feedback from the project group members (1 psychiatrist, 3 experiential experts on depression, and 2 senior researchers) and an exploratory interview with a Depression Connect member. As shown in Textbox 1, the following topics guided the interviews: (1) forum use (why, when, and how), (2) Depression Connect benefits and downsides, (3) Depression Connect working mechanisms, and (4) (relationship with) the use of other forms of formal or informal depression support and care. The complete interview guide is available in Multimedia Appendix 1. Based on interim analyses conducted after four and eight interviews, DS and AD reviewed the topic list and incorporated newly identified themes. First, we formulated new questions inquiring into the perceived associations between forum use and personal recovery (coping with depression in daily life), social recovery (effect of social ties and activities), and clinical recovery. Second, to further delineate the effects of Depression Connect use, we added questions about the development and deployment of participation styles. The adjusted topic list was then used for data collection in the successive interviews [60].

Data collection took place during the COVID-19 pandemic. The Netherlands was in the early stages of the COVID-19 outbreak when we conducted the first three face-to-face interviews. Consistent with the national measures at the time, the interviewer and participants washed their hands and maintained a physical distance of 5 feet. When new COVID-19 measures stipulated that social contact be limited, the subsequent 12 interviews were conducted via video calls. All interviews were audio-recorded and transcribed verbatim, omitting any potentially identifying data.

**Depression Connect interview themes and subthemes.**

- Use of the online community
  - Reason(s) for subscribing
  - When, why, and how is Depression Connect used
- Merits and demerits
  - Effect of Depression Connect on coping and living with depression: practical skills, meaning-giving, personal development (self-reflection)
- Working mechanisms
  - Ways in which Depression Connect as an online community and peer support method exerts its effects
- Context: other support or care
  - Ways in which Depression Connect as an online community and peer support method exerts its effects

### Analysis Strategy

The data were analyzed in ATLAS.ti (version 8.4; Scientific Software Development GmbH). Given our deductive–inductive approach, coding was both concept-driven and open. For the deductive analysis, we prepared a priori thematic codes capturing relevant themes based on the research aim and topic list. To allow findings to emerge from frequent themes without restraints imposed by predetermined concepts [58], we used open, axial, and selective coding in the inductive analysis [56,57]. To avoid a very narrow perspective, each interview started on an open-coded basis. The data were disassembled into fragments, which were compared with each other and grouped into subject categories. We used a hierarchical category system (eg, a tree diagram [57] to indicate subordinate and parallel codes and categories. When no new open codes were necessary to cover the data, axial coding was initiated. This more abstract process was used to find connections between and among categories and give coherence to the emerging analysis. Dominant and less-important elements in the data were determined to allow selective coding. At that point, the inductive and deductive approaches were combined by harmonizing the category system (based on open and axial coding) with the predetermined concepts (eg, empowerment) [51]. Categories were thus organized and integrated to uncover relationships between user engagement, Depression Connect appreciation, and the working mechanisms Depression Connect members had proposed. An open network, not specifically indicating causal linkages [58], was developed in which all the data, including the negative cases, was described and interpreted.

To ensure interrater reliability, authors DS and AD met at each stage of the process to discuss codes and themes and resolve any discrepancies. Coding was performed by an independent researcher experienced in qualitative research but not involved in the research project. The small intercoder variance was resolved by analyzing the coded segments collectively. To increase analytic sensitivity, inconsistently coded blocks were segmented into smaller units and awarded a more specific code, accompanied with a definition that included criteria for the coding of similar segments [61]. Potential interviewer or researcher bias was reduced by having participants check the outcomes to validate and verify the interim and end results. At the first member check after seven interviews, we sent all 7 participants a synthesized summary of the data analyzed thus far by email to verify whether the results resonated with their individual experiences. Participants were asked to read, comment, and return the forms. We used nonscientific wording and open questions, leaving room for individual feedback. Six participants returned the forms, and their responses were incorporated into the data set to match this data to the open network [62]. At the second member check after the final interview, we sent all 15 participants a report of the interim results together with an invitation to discuss the report per email, individual video call, or telephone. Three participants responded, providing feedback via individual video calls. Together, this enabled us to fine-tune the terminology in the interim and final results. Finally, to increase validity and to ensure any new insights into the concepts and results would be taken into account, authors DS and AD maintained a logbook in which they shared personal and theoretical views related to the research and interpretation of the data.

Participants were anonymized and identified by a randomized number (P1, P2, etc), their gender, and age. Below, we present anonymized quotes from participants to illustrate emergent themes.

## Results

The interviews provided rich data covering many aspects of engagement on Depression Connect and its perceived benefits and drawbacks. We have presented the results in the order in which topics were addressed, starting with the participants’ reasons to subscribe, followed by participation styles, and user valuation. We then describe the associations observed between participation styles and the perceived value of Depression Connect. Next, we summarize the negative aspects of Depression Connect use and, finally, discuss the use of Depression Connect in relation to face-to-face support, social networks, and mental health care.

### Participants’ Reasons to Subscribe

Given their persisting symptoms, the participants were at a stage of learning to cope and live with depression in the longer term with a focus on rehabilitation (except for 1 participant who was first diagnosed with depression 6 weeks before the interview). A total of 13 (87%) participants described a sense of loneliness or lack of social support as the main reason to engage in the online community. Their primary objective was to look for support in living with depression, which was described as a need for recognition and a genuine understanding from peers:

I feel quite lonely in this world. At home, it’s difficult for me to speak openly about my problems. When I use the online community, I come into contact with like-minded people. Usually, for tips or a “pat on the back,” things I miss at home.

P11, male, 55 years

One participant (negative case) emphasized this finding, while she did experience social support in daily life and unsubscribed from Depression Connect.

### Participation Styles

The participants used three different participation styles: *reading* messages of peers, *posting* messages to share experiences and ask questions about (coping with) depression, and *responding* to experiences or questions of other users in order to support them. Two female participants did not post any messages because they had issues with sharing personal information. One male user did not post any responses because he struggled empathizing with fellow users.

The data show that the deployment of a specific participation style was dependent on the participants’ current mood or state of mind. When feeling low, users mainly read posts or posted messages but did not respond to others’ input. Overall, after joining Depression Connect, most participants first looked for support and recognition by reading the experiences from peers and posting questions about handling the illness or writing down their own story. Gradually, when their mood had improved or when they felt more at home with or committed to the Depression Connect community, participants felt more able to support their peers and started responding to others. A user’s participation style could vary within a single session or differ per session, with their engagement on the forum generally developing from reading only to posting, and eventually responding:

At first, I thought people were just nagging a lot in their messages on the forum. I was trying to focus on solving my own problems until I saw that users were helping each other. I realized I could also benefit from their support. I began typing up my personal story. I got positive replies and then also started to respond to others.

P14, female, 62 years

### User Valuation

#### Overview

In general, the participants did not report any improvements in depressive symptoms directly associated with the use of Depression Connect but often spoke of a process toward accepting the long-term nature of their depression. Hence, the values of Depression Connect lay more in the social, emotional, and practical support in learning how to manage the illness:

It feels good when I find recognition in the messages of others. It doesn’t mean I no longer feel depressed. It just has a positive effect. Also, I get new ideas about treatment options, for example, which will eventually have a positive impact on my symptoms.

P12, female, 47 years

The positive effects the 15 users associated with their use of Depression Connect can be clustered into four main themes. Ranked according to their importance, these include a sense of belonging, emotional growth, self-efficacy, and empowerment.

#### Sense of Belonging

Most participants reported that the main benefit of Depression Connect use was the sense of belonging it provided. Recognition, emotional support, and more intrinsic understanding from peers corresponded to their reasons to subscribe, such as loneliness or a lack of support in coping with depression:

It feels like a warmhearted environment. You feel connected with people through recognition. Other users recognized the feelings I’m struggling with. In turn, I recognized the struggles of others in expressing and sharing their emotions. It all contributed to a natural sense of connectedness, which grew very fast. It feels like I’m in the right place.

P2, male, 65 years

Two participants (negative cases) did not derive a sense of belonging from the online community because they did not aim for social support; one of them felt sufficiently supported by face-to-face peer contact and the other, by her offline social network.

#### Emotional Growth

The data further showed Depression Connect to function as a tool for emotional growth: most participants saw Depression Connect use as an incentive to develop and reflect upon personal coping skills and ideas about the (longer-term) management of their depression. Although some topics they read about directly created a sense of recognition for a few participants (which was associated with a sense of belonging), other issues did not directly relate to them but often did trigger them into reflecting on the role the issue might or should play in the management of their depression. This process of personal identification raised the users’ (self-)awareness, a necessity for the development of self-knowledge and encouragement for emotional growth. Their narratives indicated that the various processes of self-reflection encouraged them to put their problems into perspective, promoting emotion regulation:

Well, when you’re sharing experiences you get different viewpoint and more insight, this makes you think more seriously, like, “Ah, that could be the same for me, or maybe that’s a pitfall for me too.” Quiet introspection can help make things more clear and may even be very helpful.

P7, female, 53 years

Maintaining online contact with peers did not solely serve as an incentive to reflect upon management and coping strategies. In and of itself, peer contact also helped users develop (better) communication skills. Participation on Depression Connect lowered the threshold to talk (ie, post) about depression; for some participants, the forum also served as a place to practice opening up about depression in face-to-face contacts. Moreover, disclosures tended to invite peers to challenge their negative-thinking patterns. In this context, adopting a relatively mild attitude toward oneself was mentioned as an important aspect of information sharing:

Maybe, it’ll also become easier to speak openly to people in person. I think it’s important to practice first, to really get the sense that I’m able to open up before actually doing so in more difficult situations.

P12, female, 47 years

Since the two negative cases did not mention emotional growth, we speculate that Depression Connect users need to experience a sense of belonging (which they also said they lacked) before they could benefit emotionally from their contact with peers.

#### Self-Efficacy

Most participants derived a greater sense of self-efficacy in coping with depression from the online community. Being informed or reminded about (other) coping mechanisms seemed to contribute to their sense of autonomy. Given the longer-term nature of their symptoms, users appreciated tips and experiences about specific treatments, medications, and publications on (coping with) depression the most. About half of the participants (7/15, 47%) also valued more practical advice, using the tips and recommendations about everyday activities as an incentive to (re)engage in these so-called self-management strategies, such as going for a walk or performing relaxation exercises:

Sometimes I read messages other users post, like “I really have to go outside more, but I don’t want to,” and then, a few hours later, the same user wrote “Actually, I went for a bike ride.” That is when I think, “Yeah, I have to go outside too [laughs].” So yes, I have to admit, reading such posts can be an incentive. Also, certain books that people mention can make me curious, prompting me to look for more information. But it depends on how people write about things. When they share information about coping strategies, I “cherry-pick” the things that suit me most.

P8, female, 61 years

#### Empowerment

Besides increasing their sense of self-efficacy or, more specifically, autonomy, the data suggest that participating in the online community empowered most participants to come to terms with and manage their depression, with three-fourths (11/15, 73%) of our participants describing Depression Connect as a tool to provide meaning to their experiences. They explained that being of value to others living with depression and supporting peers through sharing their own experiences, provided them with a (great) sense of fulfillment:

What I try to convey is: Maybe you don’t have any perspective now, I understand, I felt the same: “What am I doing here, on this planet?” But it will pass, really, it will pass. Even when the response is just a “thank you,” it gives me fulfillment.

P8, female, 61 years

After all the problems they were facing because of their depression, the users felt that participating in Depression Connect *finally* afforded them a positive and valuable experience. This seems to enhance the belief in their own strength:

You don’t get stuck in fear. For example, when you have anxieties or feel depressed, you can feel helpless, you feel lost. When you read messages of your peers saying, “It will pass,” it’s like, “Yes, it will.” This way you encourage yourself to adopt a different attitude toward depression. And, as a consequence, when you get to feel more in balance, you can support others too.

P10, male, 62 years

Consistent with their missing a genuine sense of connectedness with their Depression Connect peers, 2 participants (both negative cases) did not report deriving fulfillment from being of value to others.

### Participation Styles and the Perceived Value of Depression Connect

#### Overview

**Figure 2:**
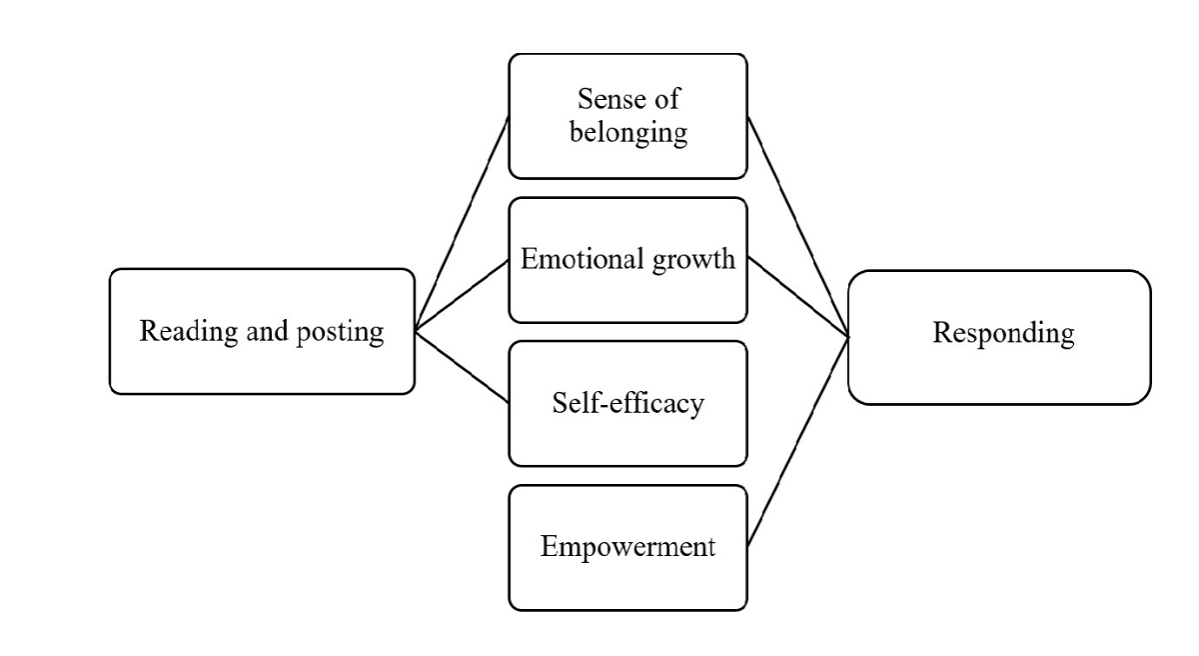
Participation styles and the perceived value of Depression Connect.

To determine how and why Depression Connect users rated the merits of forum participation, we analyzed the interaction and synergy between their participation styles and valuations. The data (schematically depicted in Figure 2) suggest that reading and posting—the styles most users restricted themselves to initially—contribute to a sense of belonging, emotional growth, and self-efficacy, whereas responding, which they later engaged in, was more likely to promote empowerment in addition to a sense of belonging and emotional growth. The elucidation and participant quotes below illustrate this relationship.

#### Reading

By reading others’ posts, users learned they were not alone in their struggle to learn to cope with negative feelings, recognition of which promoted a sense of belonging:

When others wrote about the difficulties at work they experienced on account of their depression, for instance. Suddenly, there was this recognition. A positive sensation because it made me feel like “Ah, I’m not the only one!”; it’s like I read what I could have written myself…It’s reassuring. Like in, company in distress makes trouble less.

P12, female, 47 years

Furthermore, reading their peers’ experiences made users reflect on what the topics meant for them personally, furthering their emotional growth. Finally, the practical tips helped them apply (new) coping skills in their daily lives, which enhanced their sense of self-efficacy.

#### Posting

In general, posting played a significant and positive role in the appreciation of the forum. Writing down their feelings and struggles in managing their depression often offered users relief, whereas peer recognition and understanding or merely the knowledge that their posts were read by others was of (great) value to Depression Connect users, contributing to their development of a sense of belonging:

The online community serves as a “lifeline” for me. Several times, when I was really struggling, I posted a message on DC [Depression Connect]. Not to get a response, but primarily to be able to express myself by writing down my feelings. I also write for myself, to give words to my emotions. However, writing on the forum differs because I know my posts are being read. Actually, most of the time, people even respond. It’s mainly the recognition they articulate that affects me, in a positive sense. Which in itself is quite strange because the recognition of others doesn’t essentially change how I feel. But apparently, it works. In the sense that it sort of works as a “lifeline.” A couple of times when I was doing terrible in the morning and I posted something, it was the responses of others that helped me get through the day.

P3, male, 48 years

Moreover, participants explained that sharing their personal story was healing. They managed to organize their thoughts when writing, often reinforcing self-reflection and emotional growth:

Writing about my emotions gives me peace of mind. The negative feelings don’t disappear completely, but I’m better able to dissociate myself from my problems. What I get from when other users respond to my post is a sense of “not being alone in this world.” More people are struggling with these same problems, who even try to help others. … It helps me put things into perspective, makes my problems feel less overwhelming.

P4, male, 31 years

Finally, posting specific questions about coping with depression often prompted practical tips and other new information, which is likely to have fostered a (greater) sense of self-efficacy.

#### Responding

Users responding to others’ posts derived emotional support and recognition from their peers, which strengthened their sense of belonging. Since providing support or advice entailed having to write down one’s thoughts and thus reflect on one’s own experiences, this interactive participation style seemed to promote emotional growth. Similar to posting, communicating with peers helped users to better organize and formulate their thoughts:

Yes, yes, and I think that’s exactly what the online community contributes: learning to think about, learning to reflect on yourself in other, somewhat different contexts. The things you're saying to the other are actually the things that you would like to say to yourself at that moment. Yes, and maybe that’s precisely what you do, unconsciously? When you're able to sort of put yourself in the emotional world of another, then you actually feel how good it is to connect with your own emotions. This is when I realize that that is the ultimate goal.

P2, male, 65 years

Moreover, responding and helping peers raised the users’ sense of fulfillment, which fostered a greater sense of empowerment because they felt they were of value to others.

### Negative Aspects of Depression Connect Use

As to the negative aspects of Depression Connect use, these are best captured under the notion “getting too caught up.” Participants explained they could become overwhelmed by the sheer volume of information appearing on the online community, the pressure of having to be continuously available and the stress caused by the concerns they had about the worries of their peers:

Well, you can feel overwhelmed by it all. I had mixed feelings. On the one hand, I felt relief because I could share my experiences. But on the other hand – since I visited DC [Depression Connect] a few times a day, and partly because of all the notifications I received, all the new posts – I thought, “This is not good. I’m too preoccupied with the forum and worry too much about others right now.”

P8, female, 61 years

Users also did not appreciate the forum when their messages appeared to be misinterpreted or when they received unsolicited advice. Although, as mentioned above, half of the participants valued the practical tips on coping with depression, the other half could get frustrated because they felt they were “already aware” of the recommended strategies, or it aggravated their self-criticism because they failed to engage in the suggested activities:

In itself, it was good advice, definitely well-intentioned. Also, the content was completely accurate, but I was unable to follow up on it. I felt frustrated because I agreed and knew it was sane advice, it would be the sensible thing to do, but I just couldn’t.

P3, male, 48 years

Moreover, participants reported that some members confused their own experiences and emotional needs with the personal and unique needs of peers, resulting in useless feedback and a general sense of lack of support.

### Use of Depression Connect in Relation to Face-to-Face Support, Social Networks, and Mental Health Care

In general, the participants characterized the use of the online community as being complementary to their real-life peer contacts, their social network, and any professional care or treatment. As they did not feel judged by their Depression Connect peers, the participants referred to the online community as “an emotionally safe context.” Not wanting to (over)burden their family and friends with their troubles, initially sharing feelings and receiving peer support online was helpful to some degree:

It’s about the feelings you share; we’re all struggling with depression. It’s different from friends of mine who also suffered from depression and are the most approachable people in my network, where I sometimes think, “I don’t want to bother them with my complaints again.” This is much more anonymous. It is voluntary, which is nice because a friend can try to be too supportive and say, “I’ll come and see you tomorrow,” where I think “You don’t have to come, I only felt like sharing my thoughts because I was having a bad day.” Obviously, things like that don’t happen in an online community like this.

P12, female, 47 years

At a later stage, Depression Connect interactions served as an exercise for self-disclosure in the offline world. Furthermore, anonymous participation, the voluntary nature of Depression Connect engagement, and its 24/7 availability were also mentioned as distinctive positive features compared to seeking or receiving face-to-face support via social networks or from mental health professionals:

The fact that you can log on day and night, that’s its great strength. As opposed to my psychiatrist, whom I can’t email in the middle of the night. I mean, I can, but there’s no response.

P9, female, 42 years

The overarching principle in the relationship between the use of Depression Connect and other forms of support for depression appeared to be the opportunity the forum offered to reflect on and practice the (social) skills the users were trying to master in their daily lives or through psychotherapy. Specifically, discussing topics concerning social interactions and behavioral patterns with peers were considered beneficial:

I see the online community as a stepping stone for real-life social interactions with others. I learn by writing down how I should respond, how others might respond. So I’m practicing and learning. Also, I’m learning to become more self-confident so that I can connect better with others.

P4, male, 31 years

When I’m doing schema therapy with my therapist, difficult issues come to light. I found it helpful to write about these difficulties. It allows me to reflect a bit more on them, and on top of that, I can get some advice.

P9, female, 42 years

## Discussion

### Principal Findings

#### Central Aim

In light of the promising evidence for depression ISGs [3,11,63,64], we evaluated the relevance of Depression Connect, a newly launched online peer support community based on interviews with a selection of its users. In line with previous research [16,22], we expected that the user narratives would reflect improved empowerment. Given its central role in (online) peer support [39-41] and to clarify the concept [42,46,47], we explored the purport of its constituent constructs, and, most importantly, the role different styles of user engagement played in the users’ evaluation of Depression Connect.

#### Perceived Value, Participation Styles, and a Central Drawback of Depression Connect Use

Participation in the online community engendered a sense of belonging and promoted the users’ emotional growth and sense of self-efficacy and empowerment, with self-efficacy and empowerment boosting their sense of autonomy. Where improved empowerment mainly pertained to interactional and behavioral constructs [48,49], such as meaning-giving and being of value to peers through providing support, gains in self-efficacy mostly concerned intrapersonal constructs such as being informed about treatments. With respect to modes of user engagement, three styles were identified, starting with reading only, evolving into posting, and culminating into responding. Individually and together, these participation styles related differentially to the users’ (overall positive) appreciation of the platform. As a truly interactive engagement style, *responding* played a key role in empowering users, and being valuable to others boosted their belief in their own abilities (personal strength). Primarily, the participants used the forum to explore and try (new) coping and social skills for later use in their real lives. The central drawback of Depression Connect use was that some users had become too involved in the community, getting overwhelmed by the continuous supply of posts and messages and their empathy for their fellow users. Finally, they noted that the Depression Connect community had provided them with an emotionally safe context to reach out to others in addition to their seeking or receiving face-to-face support and professional care.

#### Empowerment

One definition of empowerment in the context of this study reads “health care professionals collaborating with patients to help them acquire knowledge and resources” [46], which implies that it requires an inherently unequal relationship—one between knowledgeable health professionals and uninformed patients—to acquire knowledge and skills in managing a condition. Because of this paternalistic interpretation, the construct of empowerment is being criticized, as it contradicts the collaborative nature of the process [47]. Together with earlier positive findings on ISGs [14,18,19], our results suggest that offline and online peer communities for depression can be quite helpful for users to learn about and try new management and coping techniques. The reciprocal and “same-level” character of peer support defies the paternalistic notion of empowerment [47]. In terms of empowering patients, interactions with peers may even supplement professional care given that sharing experiential knowledge is not part of the therapeutic relationship.

Considering empowerment is a process rather than a mere outcome [46,49], we found that use of the Depression Connect platform specifically supported processes such as helping others [43] and meaning-giving. Peer contacts, and particularly sharing experiential knowledge to support others, fostered an external focus, consistent with the assumption that ISGs promote interactional empowerment. As an integral part of the process toward empowerment [46], we found that self-efficacy was mainly boosted by intrapersonal processes (ie, gaining personalized information on depression and coping skills) mirroring intrapersonal empowerment. Accordingly, we presume that participating in ISGs helps advance both intrapersonal and interpersonal or interactional empowerment.

### Findings in Context

#### Development and Variation in User Engagement

The benefits the Depression Connect users we interviewed derived from the forum are consistent with findings of other studies: informational and emotional support [11,12,14-17], social companionship [11,26], and empowerment [16,22]. Exploring which mechanisms drive ISGs and Depression Connect in particular, we compared styles of user engagement with the users’ judgments. Although the three participation styles we identified (reading, posting, and responding) all had their own merits, the users’ narratives revealed differential patterns in their online behavior. As alluded to in the introduction, previous ISG studies generally distinguished “lurkers” (ie, readers) and “posters,” that is, users with fixed behavior patterns [43]. However, our results suggest that due to the cyclical and erratic nature of depression participation styles tend to evolve and fluctuate. According to most participants, the autonomy in choosing how they engaged in Depression Connect was a core advantage of online peer support, distinguishing it from other forms of offline peer support or formal care. When faced with (recurrent) depression, people often feel compelled to keep functioning well in daily life, being a good spouse, mother or father, employee, friend, or even patient [51]. When seeking support online, they do not feel this pressure and can let themselves be guided by their current needs. Whether they translate this behavior and positive experience to everyday life remains unknown.

Moreover, the development of and variations in participation styles over time contributed to user satisfaction. After a passive start, users gained more confidence from reading others’ posts and responses and became more (inter)active themselves, making the shift from reading only to asking for help, sharing experiences, and finally helping others. Posting and responding brought gratification, boosting the way they thought about themselves, adding to their self-confidence, which Schwartz termed the “response shift effect in peer support” [65]. Nevertheless, future investigations should confirm whether accessible online communities like Depression Connect facilitate the transfer of learned skills to daily life.

#### Participation Styles and Perceived Value of Online Peer Support

In addition to the development of and flexibility in user engagement over time, our data suggest a direct association between participation styles and the perceived value of Depression Connect as an online community, which expands the findings on depression ISG research [19]. We found that the hypothesized relations between participation modes and ISG appreciation are similar to processes and associations observed in mental health care. Thus, the relationship between responders and enhanced empowerment resembles the benefits people derive from the so-called “helper role” [65] during group sessions or peer support meetings. The positive effects of helping others by responding to their narratives, such as feeling useful [66,67], promotes empowerment, as is also reflected by the growing (self-)confidence Depression Connect users reported when they began responding to peers. The observed association between posting and emotional growth or emotion regulation (ie, increasing self-knowledge through reflection on coping processes) echoes the role of expressive writing in reducing psychological distress [27,68]. By posting, simply another form of expressive writing, Depression Connect users found themselves learning to express and control their emotions better. In sum, our findings show that ISG members use passive, active or interactive styles of engagement to seek and derive different types of support from online peer communities, dependent on their personal needs over time.

### Practical Implications of ISG Use

In their systematic review, Leamy et al [30] pose that in the context of recovery-oriented mental health care, coping with depression exceeds self-management and clinical recovery. They propose important themes for personal recovery, including connectedness and empowerment [30], which correspond to the main advantages mentioned by Depression Connect users in our study. Hence, we posit that participation in an ISG may facilitate and possibly accelerate recovery (ie, improved symptom management), with users finding their own paths. Importantly, we found that the Depression Connect platform was mainly used in addition to professional psychological or psychopharmacological care, experiences with which were exchanged, with peers offering participants different, experiential perspectives on (coping with) depression. Since ISGs offer its members a more holistic approach to their mental health issues and associated problems, health professionals may consider recommending them to (some of) their clients to complement ongoing therapy or as a form of informal follow-up care after therapy discontinuation. As a matter, of course, they are advised to inform themselves and their clients of the potential adverse events associated with online fora [69].

### Limitations

Depression Connect users we interviewed may not be representative of all Depression Connect members; apart from the 2 negative cases, most participants were probably among the more frequent users because they were the more likely to come across the invitation for participation we posted. Furthermore, because the interviews were conducted during the COVID-19 pandemic when face-to-face contact was restricted, the importance of online types of support for depression increased, potentially causing the results to be biased in a positive direction.

The high accessibility (ie, free and easy of use) of the Depression Connect platform, the encouraging but nondirective role of its moderators, and its structural embedding in both a patient and mental health organization may have fostered social and interactive processes (eg, connectedness and support) that may not be representative of other ISGs that are less closely monitored [70]. Moreover, since Depression Connect is a Dutch-language forum and all participants were Dutch, we do not know whether our findings can be generalized to ISGs in other countries. It is possible that Dutch users attribute a greater value to (online) peer support because such services are not embedded in regular depression care in contrast to other countries, such as Germany [71]. Finally, the benefits our participants claimed to derive from the use of Depression Connect largely reflect short-term gains, as the duration of their forum participation varied from 1.5 to 11 months at the time of data analysis.

### Future Research

In a quantitative parallel study, we are in the process of evaluating the effects of Depression Connect use on empowerment (primary outcome measure) after 3 and 6 months. Further longitudinal research should be aimed at the longer-term beneficial and adverse effects of participation in ISGs.

A mixed-method effectiveness study should address the complexity and potential of peer support interventions. The method can yield rich and comprehensive data and thus provide a more holistic view on how people cope with depression. In this context, examining the perceived level of social support in daily life in relation to user statistics of online peer support services will be informative. Finally, a key challenge is to determine whether skills learned from peers in online networks also contribute to mental health recovery in the offline world [72].

### Conclusions

Users of Depression Connect considered the online peer support community an accessible and valuable tool for learning to cope (better) with their depression. Seeking to understand the working mechanisms of ISGs, we found that the greater majority of the study participants benefited from the freedom and flexibility Depression Connect offered, allowing them to employ passive, active, and interactive styles of user engagement depending on their current mood and needs. Most found the forum, monitored by experienced peers, a safe environment to practice social and coping skills for later deployment in the offline world, supplementing formal and informal care. We found that besides promoting intrapersonal empowerment, Depression Connect also fostered interactional empowerment. Provided platforms are closely monitored and used to complement or follow-up formal care, and pending further investigations, we suggest that online peer support may be recommended as a safe context for exchanging knowledge and experiences on how to cope with depression and practice newly gained insights and skills.

## Appendix

Multimedia Appendix 1 Interview guide.

## Abbreviations

ISG: internet support group
MHISG: mental health internet support group
RCT: randomized controlled trial
