# Supplementary material for: A Newly Developed Online Peer Support Community for Depression (Depression Connect): Qualitative Study
Source: J Med Internet Res. 2021 Jul 12;23(7):e25917. doi: 10.2196/25917 (PMC8314160; doi:10.2196/25917)
Supplement: Multimedia Appendix 1 [file jmir_v23i7e25917_app1.pdf]

Interview guide: Qualitative evaluation of the online peer support community "Depression Connect" (DC)
By: Dorien Smit and Amber Dings

**Introduction**	Introduce yourself<br>Explain<br>• the rationale and objectives of the study (and research project)<br>• anonymity, confidentiality, voluntary participation; possibility to ask for a recess or end the interview at any time<br>• your role as researcher<br>• audio recording<br>• expected duration: 45 minutes<br>• informed consent
**Background**	Screening and brief synopsis of the interview<br>Any questions/comments?
**Reasons for signing up with DC**	When and why did you subscribe to DC?<br>What was your aim when you first joined DC?
**Forum use in general**	In general, **when** do you use the forum, **why**, i.e. with what **objective**?<br>*For example: did you have a specific question/need for information or simply out of general interest?*
**Merits**<br><br>**Participation style(s)**	Can you give an **example** when you benefited from forum use?<br><br>When is the forum (most) useful?<br>- For **what** goals<br>- **When** (under which circumstances)<br>- In **which state of mind**? In a more critical and/or stable phase of depression?<br><br>Why opt for this online forum; in what way(s) does it **differ** from other options *(e.g. professional help, social support in daily life)*?<br>How would you describe the **usefulness** of the messages of other users?<br>Do you think/feel you can **help or support** other DC users?<br>Would you recommend DC and, if so, to **whom** would you recommend it?<br><br>**Personal/social/clinical recovery:** To what extent do you benefit from DC in coping with your depression on a daily basis?
**Demerits**	Can you give an example when you did not benefit from the forum?<br><br>**When,** for **whom** or under **which circumstances** is the forum **not useful?**<br>**Why?**
**Current role/relevance of DC**	What is the role/relevance of DC in your life at this moment?
**Role DC in relation to other means of (in)formal support**	What is the role of DC in relation to other informal support and/or formal care for depression?<br>- Social network<br>- Mental health care (psychological/psychopharmacological treatment)<br>- Live peer support<br><br>Have you benefited from DC use in ways you had not expected?
**Open question**	Do you have something to add, topics we did not discuss that you think are relevant to characterize the (dis)advantages of Depression Connect?

**Concluding the interview**	Are you willing to participate in an interim assessment and provide feedback on the interim results?<br>Thank the participant for his/her contributions
